# A new approach for determination of orthophosphate based on mixed valent molybdenum oxide/poly 1,2-diaminoanthraquinone in seawater

**DOI:** 10.1038/s41598-023-40479-w

**Published:** 2023-08-21

**Authors:** Mahmoud Fatehy Altahan, Magdi AbdelAzzem

**Affiliations:** 1 Central Laboratory for Environmental Quality Monitoring, National Water Research Center, El-Qanater El-Khairia, 13621 Egypt; 2 Electrochemistry Laboratory, Chemistry Department, Faculty of Science, Menoufia University, Shibin El-Kom, 32511 Egypt

**Keywords:** Environmental monitoring, Marine chemistry

## Abstract

Orthophosphate is an essential macronutrient in natural water that controls primary production and strongly influences the global ocean carbon cycle. Electrochemical determination of orthophosphate is highly recommended because electrochemistry provides the simplest means of determination. Here the determination of orthophosphate based on the formation of a phosphomolybdate complex is reported. Mixed-valent molybdenum oxide (Mo_x_O_y_) was prepared by cyclic voltammetry on poly-1,2-diaminoanthraquinone (1,2-DAAQ), which was performed by cyclic voltammetry on the surface of a glassy carbon electrode under pre-optimized conditions for the thickness of the modified electrode layers. The proposed modified electrode was used for square-wave voltammetry of orthophosphate ions under pre-optimized square-wave parameters (i.e., frequency and amplitude) in strongly acidic medium (pH < 1). The linear range was 0.05–4 µM with a limit of quantification (LOD) of 0.0093 µM with no effect on two peaks due to cross interference from silicate. Furthermore, Mo_x_O_y_/PDAAQ shows good reproducibility with a relative standard deviation (RSD) of 2.17% for the peak at 0.035 V and 3.56% for the peak at 0.2 V. Real seawater samples were also analyzed for PO_4_^3−^ analysis by UV spectrophotometry and the results were compared with the measurement results of our proposed electrode, with good recoveries obtained.

## Introduction

Phosphate is considered as an essential chemical compound in the body's cells. Phosphate plays an important role as a building block for phospholipids, nucleic acid, and adenosine triphosphate (ATP). Those biomolecules play an important role in the storage or utilization of genetic information^1,2^. However, when excessively accumulated, phosphate can cause many serious social problems, which are reflected in its strong environmental and health effects^3^. In the environment, phosphorus can be occurred in different chemical forms, which can be classified as follows: inorganic phosphates such as orthophosphate (PO_4_^3−^), which is considered the usual soluble form of phosphate. Other condensed phosphate forms are pyro- (P_2_O_7_^4−^), meta- (PO_4_^3−^) and polyphosphates ([PO_4_^3−^]_n_) and the second form is organic phosphates^4^. Most of the inorganic phosphates are contained either in agricultural fertilizers or in food. Those phosphate forms enter the environment and lead to eutrophication of surface waters. Those are causing extreme production of biological material (mainly algae) and throttling of aquatic ecology^5^. In addition, excess or deficiency of phosphate can cause many serious human diseases such as hypophosphatemia (phosphate deficiency) and hyperphosphatemia (phosphate excess)^6^.

It is obvious that a highly sensitive, stable, and safe determination tool is needed for continuous monitoring of phosphate to detect its environmental and health effects. For example, the colorimetric method is still the recommended standard method for measuring phosphate in water. The method incorporated molybdenum ions which are complexed using antimony tartrate (C_12_H_12_O_18_Sb_2_) and ascorbic acid as reducing agents to form yellow or blue phosphomolybdate [PMo_12_O_40_]^3−^ or [PMo_12_O_40_]^4−^, respectively^7^. This method, based on enormous reagents, is time consuming and cannot be used for on-line measurements. Alternative analytical techniques for phosphate detection have been developed, such as ion chromatography (IC), luminescence/fluorescence sensors, and biosensor^8^. Nevertheless, electrochemical determination methods have already been developed using mobile devices^9^.

Electroanalytical methods developed for the determination of phosphate offer many advantages such as rapidity, cost efficiency, and lack of intervention due to sample turbidity. In the last decades, many potentiometric ion-selective electrodes (PISE) have been developed based on either a metal, organic complexes or metal complexes^8^. Various forms of PIES have been investigated, with metal-based PIES such as silver phosphate sensors suffering from interference from chloride ions^10^. PIES of organic complexes such as organic tin compounds (IV)^11^, PIES of cobalt using a cobalt phosphate/Co electrode^12^, PIES of metal complexes such as Zn (II) complexes^13^, and finally PIES of polymers such as polyaniline/gold electrodes^14^. These PIES have good lifetime; however, the sensitivity is different due to some interferences that require subsequent calibration before use. Polyvinyl chloride (PVC)-based bis(dibromophenylstannyl)methane, which detects only HPO_4_^2−^ and other inorganic phosphates^15^. These PIES have not been tested in surface or contaminated water. Other voltammetric sensors developed for the determination of phosphate in water use cyclic voltammetry (CV)^16^, which is not suitable for rare nanomolar phosphate concentrations. Pulsed techniques such as differential pulse voltammetry (DPV), which has a detection limit of 0.19 µM^17^, but has some difficulties due to the complicated optimization of DPV parameters and lack of repeatability.

Another type of pulsed technique, square wave voltammetry, shows better performance compared to DPV. Square wave voltammetry was first used by Barus, using an Au electrode as the working electrode and a Mo electrode to electrolyze molybdate. A LoD of 0.05 µM was obtained^18^.

Recent work has been published on the determination of phosphate on molybdate/carbon paste electrode pretreated with Sodium chloride solution and used for square wave voltammetry of orthophosphate^19^. The application of the proposed method was also reported into a prototype based on the Bi-potentiostat technique into flow injection analysis^20^.

In this article, for the first time Mo in the form of molybdenum oxide is reported, which was previously prepared by Tian et al.^21^ and applied to poly 1,2-diaminoanthraquinone. Poly 1,2-diaminoanthraquinone along with other isomers were previously prepared by our group^22–25^. This work could help achieve our investment goal and anticipated goals of expanding the use of on-site sensors to assess water quality^26^.

This electrode is used as a fast electrochemical sensor for direct square-wave voltammetry of phosphate ions. The electrochemical measurement was performed without additives under acidic conditions. A very low detection limit with good repeatability was obtained. Good recoveries were obtained when real seawater samples were analyzed using classical spectrophotometric methods.

## Results and discussion

### Preparation of modified electrode

The anodic polymerization of DAAQ using cyclic voltammetry (Fig. 4A) was described earlier in our previous work. The cyclic voltammogram shows three primary peaks. The first primary peak is at 0.9 V, with the peak current decreasing as the number of sampling cycles increases. This peak relates to the consumption of the monomer molecules as polymerization proceeds. The other two peaks relate to polymer formation. The first peak is at 0.5 V, where the peak current increases with increasing sampling cycles. This peak represents the reduction of the monomer DAAQ to the radical anion form. The second peak, which occurs at 0.55 V, corresponds to the oxidation of the radical DAAQ anion to the dimeric state. The introduction of those substances increases the surface area of the electrode and increases the accessibility of the electrolyte to the active sites of the electrode. In the literature cyclic voltammetry is the technique used to form molybdenum oxide on the electrode surface because of the nature of the technique for consecutive oxidation and reduction steps. The formation of molybdenum oxide mixed valent (Mo_x_O_y_) was performed using cyclic voltammetry as shown in Fig. 4B. The figure shows that the highest reduction peak at − 0.48 V in the first scan cycle is due to the deposition of the reduced molybdenum species on the electrode surface. The peak corresponds to the reduction of Mo (VI) to Mo (V) and Mo (IV) supports the formation of the polymeric layer of the oxide film with mixed valence of Mo (VI) and Mo (V), while the number of scan cycles increases. The cathodic peak current decreased because of already formed oxide film on the electrode surface. There is a low significant anodic peak at − 0.22 V may be due to the oxidation of Mo (IV) to Mo (V) during the oxide film formation.

### Square wave voltammetry of PO_4_^3−^ on Mo_x_O_y_/PDAAQ modified electrode

**Figure 1:**
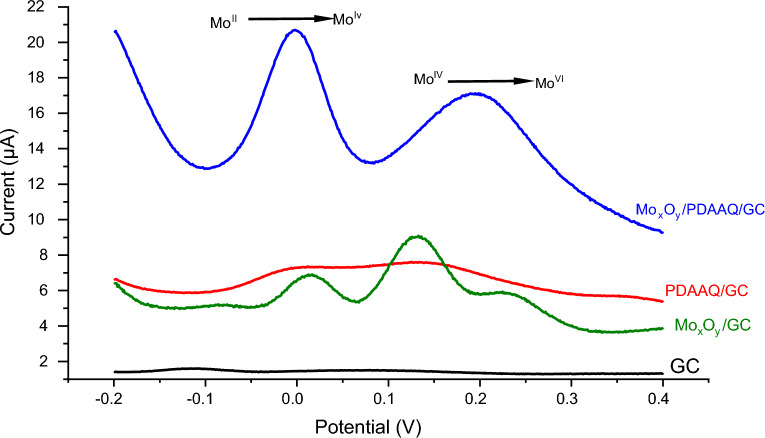
Square-wave voltammograms of 10 µM PO_4_^3−^ in a solution of artificial seawater (34.5 g/L) pH 1 with a potential range of − 0.2 to 0.4 V with a step potential of 1 mV, an amplitude of 25 mV and a frequency of 100 Hz on a glassy carbon electrode without any modification (black line), a glass carbon electrode after modification by a layer of mixed-valency molybdate oxide (green line), a glassy carbon electrode modified by a layer of poly-1,2-diaminoanthraquinone (red line) and a glassy carbon electrode modified by a layer of mixed-valent molybdate oxide and poly-1,2-diaminoanthraquinone (blue line).

Determination of phosphate ion in sea water performed using SWV at different electrode for 10 × 10^–6^ M PO_4_^3–^ (Fig. 1) firstly at glassy carbon, there is not any peak indicating the presence of phosphate ion into the solution, while with using Mo oxide film there are two clearly peaks at 0.02 V and 0.12 V are due to the gradual oxidation of Mo (II) to Mo (IV) and Mo (IV) to Mo (VI), respectively. For using the PDAAQ film, there is not any significant peak. However, at Mo_x_O_y_/PDAAQ modified electrode, two high peaks obtained at − 0.035 V and 0.2 mV with about 3 and 2 times, respectively, higher than that obtained at Mo_x_O_y_/GC electrode. For the results obtained, the MoO_3_/PDAAQ/GC is considered the perfect electrode to detect phosphate in sea water.

### Optimization of preparation parameters

One of the key parameters that has a significant impact on the performance of the conducting polymer or the metal oxides that were formed by cyclic voltammetry is the thickness of the thin film or surface layer being exhibited. The thickness of the layer of the film is investigated by the number of scanning cycles of the cyclic voltammetry. The thickness of the polymer or metal oxide film is increasing with increasing the number of scanning cycles.

The effect of thickness of polymer film on the peak currents of both peaks at − 0.015 V and 0.185 V studied depending on the number of scanning cycles during polymer formation as the concentration of the monomer remains constant during film fabrication. A solution of 10 µM PO_4_^3−^ into 30 g L^−1^ NaCl was tested with the number of scanning cycles varied from 2 to 15 cycles with 10 scanning cycles of molybdenum oxide. The results show that polymer film formed with 5 cycles is the best polymer film formed at which the highest peak current of 5 µA and 3 µA at − 0.015 V and 0.185 V are reported, respectively.

Also, the effect of the thickness of the Mo oxide film on the peak currents of the two peaks corresponding to both Mo (II) to Mo (IV) and Mo (IV) to Mo (VI), respectively, studied from 5 to 20 cycles showing that the increase in peak currents of the two peaks while increasing from 5 to 10 scanning cycles while with raising above 10 cycles, there is a steady state in the quantity of the peaks current of about 5.5 µA, 3.2 µA at − 0.015 V, 0.185 V, so that 10 scanning cycles is the preferred preparation condition in forming the oxide film electrode.

### Analytical performance of phosphate ion @ Mo_x_O_y_/PDAAQ/GC electrode

**Figure 2:**
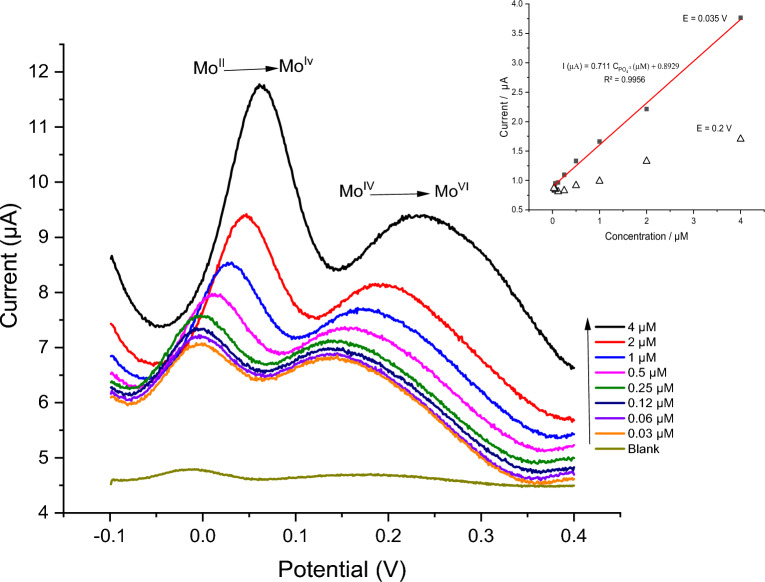
SWV of PO_4_^3–^ recorded at Mo_x_O_y_/PDAAQ/GC electrode under step potential 1 mV, amplitude 25 mV and frequency 100 Hz between − 0.1 to 0.4 V in a solution of 34.5 g/L NaCl containing phosphate ion with a concentration from 0.03 to 4 µM and the inset is the calibration curve of the current recorded at E = 0.015 V/ Ag/AgCl vs. phosphate concentrations.

A series of concentrations of phosphate ion in solution containing NaCl (34.5 g/L) as a simulated sea water used to study the analytical performance of the modified electrode (Mo_x_O_y_/ PDAAQ/GC electrode) as shown in Fig. 2, where phosphate ion concentrations varied from 0.03 to 4 µM showing a perfect gradual increasing in the peak current in accordance with raising the phosphate concentration and this clearly represented in the good linearity of the calibration plot of the current recorded at 0.015 V (while at E = 0.2 V, there is a lower value of R^2^; linearity of calibration curve) vs. the phosphate concentration with coefficient of detection R^2^ = 0.9956 and regression equation I (µA) = 0.711 C_PO4_^3–^ (µM) + 0.8929. However, with increasing the phosphate ion concentrations, there is an observed positive shift in the peak potential that could be due to the IR drop. While the LOD and the LOQ values are 0.0093 µM and 0.03 µM, respectively, which calculated using the following equations:

1 $$LOD=3\times SD/S$$

2 $$LOQ=10\times SD/S$$

where SD is the standard deviation of 10 blank responses in a solution containing only NaCl, and S is the sensitivity of the calibration curve or by another means it is the slope of the curve.

In addition, the effect of presence of some cross interferences such as silicates, ammonium, chlorides, and sulphate which usually present in real water samples were tested without any significant change in the two phosphate related peaks (Δ Ep > 1%).

Table 1 shows comparison between our state-of-the-art modified electrode and literature published modified electrode. Our modified electrode displayed superior performance for electrochemical determination of orthophosphate in the last decade, with a highly sensitive detection limit. Additionally, our method presented a simple assay compared to other published articles.

**Table 1:** Electrochemical determination of PO_4_^3–^ at different electrodes.

Electrode	Technique	Linear range (µM)	LoD (µM)	References
Mb rod^a^	Potentiometry (ISE^b^)	10–100	1.9	^27^
PANI^c^	Potentiometry (ISE)	1–100	1	^14^
Bis(DBPS)methane/PVC^d^	Potentiometry (ISME^e^)	0.5–5 × 10^3^	0.5	^15^
Chitosan-clay/PVC	Potentiometry	1–1 × 10^4^	0.6	^28^
CB-SPE-FIA^g^	Amperometry	1–80	0.1	^29^
CB/SPE	Amperometry	0.5–100	0.1	^30^
AuNWs^h^	Amperometry	12.5–1 × 10^3^	0.1	^31^
AuNWs/Pt	Amperometry	48–1.4 × 10^3^	45	^32^
Co(H_2_PO_4_)_2_/Co	Amperometry	0.1 × 10^6^–10 × 10^6^	Not detected	^33^
Au electrode	DPV^i^	0.65 × 10^6^–3.01 × 10^6^	0.19 × 10^6^	^17^
Au electrode	SWV^j^	0.1–1	0.05	^18^
Mo_x_O_y_/PDAAQ/GC	SWV	0.03–4	0.009	This study

^a^*Mb* molybdate, ^b^*ISE* ion-selective electrode, ^c^*PANI* polyaniline, ^d^*Bis(DBPS)methane/PVC* Bis (dibromophenylstannyl) methane/polyvinyl chloride, ^e^*ISME* ion-selective microelectrode, ^g^*CB-SPE-FIA* carbon black nanoparticles-screen printed electrode-flow injection analysis, ^h^*AuNWs* gold nanowires, ^i^*DPV* differential pulse voltammetry, ^j^*SWV* square wave voltammetry.

The detection of orthophosphate in seawater is crucial for understanding nutrient cycling and eutrophication processes. Various analytical methods for the detection of orthophosphate in seawater have been published in the literature, including capillary electrophoresis, photometry, spectrophotometry, ion chromatography, and others. To investigate the effectiveness of our approach, we compared it with these existing methods using Table 2. The results show that our approach outperforms the other analytical techniques in terms of the linear range, which is the typical range of orthophosphate content in seawater. Our approach showed excellent performance over the entire range of orthophosphate content, ranging from a few nanomolar in oligotrophic seawater to 3 µM in estuarine waters. This shows that our approach is very sensitive and accurate in detecting orthophosphate in seawater and provides researchers with a powerful tool to study environmental processes. Consequently, this study opens new possibilities for the detection of orthophosphate in seawater, expanding our understanding of nutrient cycling and eutrophication processes, and ultimately contributing to sustainable ocean management.

**Table 2:** Comparison between analytical methods for determination of orthophosphate.

Analytical methods	Linear range (µM)	LOD (µM)	References
Capillary electrophoresis (CE)	0.5–10	0.45	^34^
Photometric	4.8–100	0.64	^35^
Spectrophotometry (blue method assay)*	0.14–10	0.04	^36^
Spectrophotometry (yellow method assay)*	0.1–60	0.052	^37^
Ion Chromatography (IC)	1.6–806	0.76	^38^
Square wave voltammetry (Mo_x_O_y_/PDAAQ/GC)	0.03–4	0.009	This study

*The methods were applied in microfluidic Lab on Chip.

### Repeatability of modified electrode

**Figure 3:**
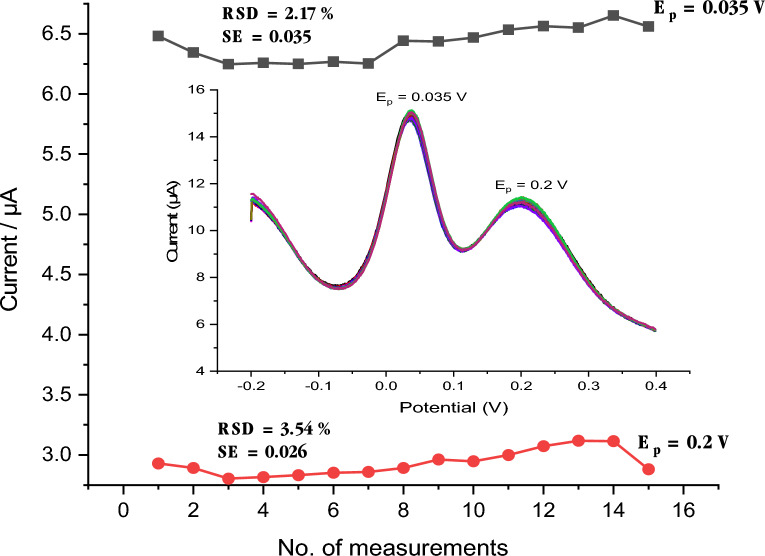
Peak current measurements of 15 repetitive measurements of 6 × 10^–6^ M PO_4_^3–^ at (Mo_x_O_y_/PDAAQ/GC) electrode in solution containing 34.5 g/L NaCl for peak at 0.035 V and peak at 0.2 V and the inset represented the data gathered from every SWV response over 15 measurements.

Fifteen repetitive measurements of the same concentration of phosphate ion (6 × 10^–6^ M) at Mo_x_O_y_/PDAAQ/GC electrode (Fig. 3) used to study the stability of the modified electrode during the repeated measurements without any significant deviation of the value of the peak current represented with RSD value of 2.17% and 3.54% and standard error (SE) of 0.035 and 0.026 for peak current at 0.035 V and 0.2 V, respectively, as shown in Fig. 3 providing the concept that our proposed method had a great advantage due to its stability during repetitive measurements.

### Analysis of real sea water samples

Real saline water samples were collected off the coast of the Mediterranean Sea (Sample 1and sample 2), certified reference materials (CRMs) termed as Reference Material for Nutrients in Seawater (RMNS). CRMs were purchased from KANSO TECHNOS CO., LTD with lot. Number CG, CI and CH. The assigned concentration of the collected samples and CRMs samples were mentioned in Table 3. Collected Samples measured by traditional colorimetric method using UV–visible spectrophotometer with the help of using Sb, ascorbic acid and in highly acidic medium (5N H_2_SO_4_). Also, the samples were analyzed with our proposed method (Mo_x_O_y_/PDAAQ/GC) electrode. Where the CRMs samples were analyzed as mentioned in certificates of Analysis (COA)^39–41^. Good recovery values were obtained using our method comparing with this well-known method ranged from 93.5 to 107%. For 5 analyses for each sample, a paired t test with a degree of freedom (df) of 4 and a significance level of 1% was used to detect systematic error (bias) between those analyzed by our approach compare with those determined spectrophotometrically. No bias was found for the Mediterranean seawater sample 1 (t-value = 0.55, t_critical (one-tail)_ value = 3.747, p > 0.01), the same is true for well water where there is no bias (t-value = 0.58, t_critical (one-tail)_ value = 3.747, p > 0.01), it is true for Kanso CRM CG (t-value = 0.38, t_critical (one-tail)_ value = 3.747, p > 0.01) and also for Kanso CRM CH (t-value = 3.44, t_critical (one-tail)_value = 3.747, p > 0.01), where there is no bias. On the other hand, significant differences were found between the values determined by our method and spectrophotometrically for Kanso CRM CI (t-value = 6.05, t_critical (one-tail)_ value = 3.747, p < 0.01). In addition, the F-test was used to determine the bias between the analyses of the samples determined by our method and those determined by the reference colorimetric method. With df between groups (df1 = 1) obtained as "k−1", where k is the number of groups equal to 2, and df2, which is the degree of freedom within the group with "n−k", where n is the total number of samples (df2 = 8). The same results were obtained as for the t-test, where no bias was obtained for the Lake Burullus sample (F-value = 8.96, F_ritical_ value = 11.259, p > 0.01), the same is true for the well water sample (F-value = 0.88, F_critical_ value = 11.259, p > 0.01) and it is also true for Kanso CRM CG (F-value = 0.15, F_critical_ value = 11.259, p > 0.01). Significant differences between the analyses of the samples by both methods exist for Kanso CRM CI (F-value = 35.7, F_critical_ value = 11.259, p < 0.01) and for Kanso CRM CI (F-value = 11.84, F_critical_ value = 11.259, p < 0.01). Overall, the analysis of real saltwater samples shows good values for three samples over 5 samples without bias observed as results of the t-test and f-test. For CRM CH, a recovery value of 97.6% above the mean values shows a good recovery value, while for CRM CI, a value of + 2 above the accepted tolerance for the recovery values (100 ± 5%) could be accepted for our approach as a substitute for the traditional method that consumes many reagents.

**Table 3:** Determination of PO_4_^3–^ at UV–visible spectrophotometer and Mo_x_O_y_/PDAAQ/GC electrode.

	Sample type	Salinity/psu	Founded by UV–visible spectrophotometer ± σ^a^ (µM)	Founded by Mo_x_O_y_/PDAAQ/GC ± σ (n^b^ = 5) (µM)	Recovery % vs. UV–visible spectrophotometer (%)
1	Mediterranean Sea sample 1	47.7	0.026 ± 0.005	0.0248 ± 0.004	95.4
2	Mediterranean Sea sample 2	52.3	0.025 ± 0.005	0.024 ± 0.005	94.5
3	Kanso CRM^c^ CG	34.338	1.70 ± 0.0011	1.67 ± 0.174	98.23
4	Kanso CRM CI	35.654	0.948 ± 0.002	1.006 ± 0.02	106
5	Kanso CRM CH	34.991	1.172 ± 0.0016	1.144 ± 0.018	97.6

^a^*σ* standard deviation, ^b^*n* number of analyses, ^c^*CRM* certified reference material.

## Conclusion

Overall, preparation of a poly-1,2-diaminoanthraquinone on a glassy carbon electrode was succeeded. Ammonium molybdate was deposited on the modified electrode. The modified electrode was used for square wave voltammetry for orthophosphate in artificial seawater under acidic conditions. A typical voltammogram for orthophosphate is obtained with two oxidation peaks at 0 V and 0.2 V. Very good linearity was obtained with an R square value of 0.9956 for a linear range from 0.03 to 4 µM with a detection limit of 0.003 µM. Good precision was obtained with an RSD value of 2.17% and 3.45% for peaks at 0.035 V and 0.2 V, respectively. Good recovery values for samples analyzed by our method compared to those measured by classical colorimetric methods. Our future work will focus on the application of our method in a fully automated prototype for on-site analysis of orthophosphate in seawater.

## Experimental

The poly-1,2-diaminoanthraquinone-modified electrode was prepared on a 3.0-mm-diameter glassy carbon (GC) electrode (Bioanalytical Systems, Inc. (BASi), USA). A three-electrode voltametric cell was used, with the glassy carbon electrode as the working electrode and the silver/silver chloride reference electrode fed with 3 M potassium chloride (KCl, Merck, USA) (BASi, USA) and a platinum wire (BASi, USA) as the counter/auxiliary electrode. All voltametric measurements were performed using an Epislon EC model potentiostat connected to a cell stand (BASi, U.S.). The glass carbon electrode was pre-washed with deionized water after being polished with a polishing set containing 0.3 µm aluminium suspension. The glass electrode was electrochemically cleaned by applying a fixed potential of 0.3 V into a solution of 0.1 M NaOH for 30 s with stirring at 400 ppm. The anodic polymerization of the 1,2-diaminoanthraquinone was done on glassy carbon followed by the polymerization of mixed valent oxide layer.

**Figure 4:**
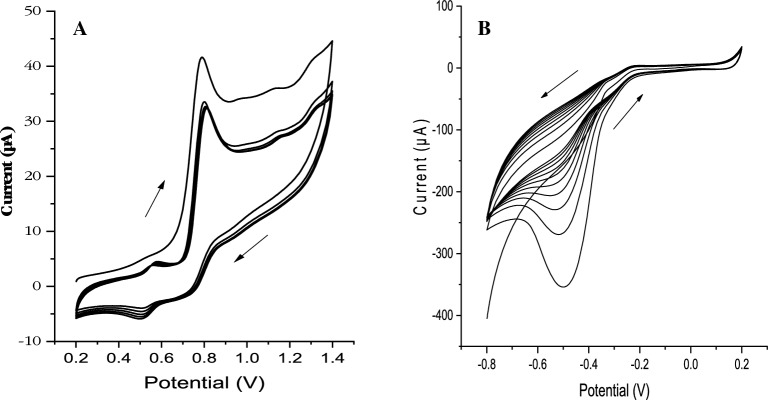
(**A**) Repetitive cyclic voltammograms of 1 mM DAAQ on glassy carbon electrode at a potential range from 0.2 to 1.4 V (vs. Ag/AgCl, 3 M KCl) at a scan rate of 0.1 V s^−1^ for five scanning cycles with increased peak current at an oxidation potential of 0.6 V and a reduction potential of 0.5 V. (**B**) Repetitive cyclic voltammograms of 5 mM ammonium molybdate tetrahydrate in a solution containing 50 mM Na_2_SO_4_, pH 3 with a potential range from – 0.8 to 0.2 V at a scan rate of 0.1 V s^−1^ for 10 scanning cycles with increased peak current at an oxidation potential of − 0.2 V and at a reduction potential of – 0.5 V.

The modified electrode was prepared by cyclic voltammetry in a voltametric cell containing 1 mM diaminoanthraquinone (DAAQ) ([C_14_H_10_N_2_O_2_], 99.99%, Sigma Aldrich, CAS no. 1758-68-5) and 0.1 M lithium perchlorate ([LiClO_4_] (99.9%, Sigma Aldrich, CAS No. 7791-03-9) in a solution of acetonitrile ([CH_3_CN] (99.5%, Merck Millipore, CAS No. 75-05-08)). Cyclic voltammetry was performed from 0.2 to 1.4 V at a sampling rate of 0.1 V s^−1^ for five sampling cycles (Fig. 4A). The electrode modified with poly-1,2-diaminoanthraquionone/glassy carbon (PDAAQ/GC) was then rinsed with deionized water and was ready for use.

The formed polymer showed very promising behavior for the chelation of metal cations which facilitate the formation of molybdenum oxide on its surface. Previously few reports have been published on the electrochemical determination of molybdenum oxide. Where the polymerization using a suitable electrolyte leads to an oxide layer with various oxidation states ranging from + 6 to + 4, where the formed molybdenum oxide electrode shows a high potential because of its high specific capacitance and good stability. The electrochemical properties of molybdenum oxide modified electrodes can be enhanced by modifying their surfaces with another active surface substances.

For the formation of molybdate oxide layer, the formed PDAAQ/GC electrode was placed in a solution of 5 mM ammonium molybdate tetrahydrate ([(NH_4_)_6_Mo_7_O_24_.4H_2_O] (≥ 99.0%, Sigma Aldrich, CAS no. 12054-85-2). Where 50 mM sodium sulphate ([Na_2_SO_4_] (9.5-100.5%, Merck Millipore, CAS no. 7757-82-6) was added with a pH of 3 adjusted with sulfuric acid ([H_2_SO_4_] (98%, Merck Millipore, CAS No. 7664-93-9). Cyclic voltammetry in the potential range of − 0.8 to 0.2 V at a sampling rate of 50 mV/S for 10 sampling cycles, obtaining the voltammogram as shown in Fig. 4B. Then, the prepared mixed (Mo_x_O_y_/PDAAQ/GC) modified electrode was washed with deionized water and was ready for phosphate determination.

Orthophosphate stock solutions with a concentration of 10 mM were prepared from di-potassium hydrogen phosphate [KH_2_PO_4_] (99.5–100.5%, Merck Millipore, CAS No. 7778-77-0). Artificial seawater was prepared at a concentration of 34.5 g/L sodium chloride ([NaCl] (99.5–100.5%, Merck Millipore, CAS No. 7647-14-5).

Square wave voltammetry (SWV) was used for the electrochemical determination of orthophosphate in seawater (34.5 g/L). SWV was selected because of its unique properties of suppressing the charging current and increasing the faradic current. It is a technique that uses a combination of square wave and stair-step potential ramp. The current is sampled twice in this technique, at the beginning of the pulse and at the end of the pulse, to reduce the influence of the capacitance current. The measurement has been done under acidic conditions into a solution of 2.5 M Sulfuric acid to achieve a pH of less than 1 to exclude the possible interferent of orthosilicate. Orthosilicate ion (SiO_4_^4–^) is the most common interferent for determination of phosphate by molybdate. It has the same tendency as orthophosphate to react with molybdate and form the silicomolybdate complex. One possible to exclude the interfering from silicate, it is performing the determination of orthophosphate at high acidic conditions at pH less than 1^19^.

Orthophosphate was determined by square-wave voltammetry in a solution of artificial seawater with a pH 1 adjusted with concentrated sulfuric acid with a potential range of − 0.2 to 0.4 V with a step potential of 1 mV, amplitude of 25 mV, and frequency of 100 mV.

Measurements on real seawater samples were performed with an ultraviolet spectrophotometer (UV) from Hach using the classic blue phosphomolybdate complex.

## Data Availability

All the data are reported within the manuscript.
